# Portrait of inflammatory response to ionizing radiation treatment

**DOI:** 10.1186/s12950-015-0058-3

**Published:** 2015-02-18

**Authors:** Federica Maria Di Maggio, Luigi Minafra, Giusi Irma Forte, Francesco Paolo Cammarata, Domenico Lio, Cristina Messa, Maria Carla Gilardi, Valentina Bravatà

**Affiliations:** Department of Pathobiology and Medical and Forensic Biotechnologies, University of Palermo, Palermo, Italy; IBFM CNR – LATO, Contrada Pietrapollastra Pisciotto, Cefalù, PA Italy; Department of Health Sciences, Tecnomed Foundation, University of Milano-Bicocca, Milan, Italy; Nuclear Medicine Center, San Gerardo Hospital, Monza, Italy; Nuclear Medicine, San Raffaele Scientific Institute, Milan, Italy

**Keywords:** Ionizing radiation, Inflammation, Cytokine, Fibrosis, Invasiveness

## Abstract

Ionizing radiation (IR) activates both pro-and anti-proliferative signal pathways producing an imbalance in cell fate decision. IR is able to regulate several genes and factors involved in cell-cycle progression, survival and/or cell death, DNA repair and inflammation modulating an intracellular radiation-dependent response. Radiation therapy can modulate anti-tumour immune responses, modifying tumour and its microenvironment. In this review, we report how IR could stimulate inflammatory factors to affect cell fate via multiple pathways, describing their roles on gene expression regulation, fibrosis and invasive processes. Understanding the complex relationship between IR, inflammation and immune responses in cancer, opens up new avenues for radiation research and therapy in order to optimize and personalize radiation therapy treatment for each patient.

## Introduction

Radiation therapy (RT) is a treatment modality used for many types of cancer: more than 50% of cancer patients receive RT, often used in combination with surgery and chemotherapy [1].

Ionizing radiation (IR) activate both pro- and anti-proliferative signal pathways altering the homeostatic balance between survival and cell death, regulated by several genes and factors involved in cell cycle progression, DNA repair, inflammation and cell death induction [2].

Studies have shown that RT may reduce the incidence of distant metastases and improve survival by controlling locoregional recurrence [3].

An increasing amount of data suggests that there is a direct relationship by which radiation stimulate the immune system, which in turn contributes to tumour cell death [4].

It has long been recognized that the immune system plays a pivotal role in tumours. On the one hand, immunological factors can suppress tumour development by killing cancer cells or inhibiting their growth. On the other hand, immune cells are able to induce an immunosuppressive microenvironment that contributes to promote tumour progression [5-7].

More precisely, inflammatory cytokines, growth factors and proteases can affect cancer cell invasion, bystander effect, radiation tissue complications such as fibrosis, genomic instability and thus can greatly affect intrinsic cellular radiosensitivity [8-10].

Recently, it has become evident that, in particular for solid tumours, the inhibition of neoplastic cell proliferative capacity following irradiation can occur through different modes of cell death that could also be induced by immunological factors (i.e. apoptosis, necrosis, mitotic catastrophe, autophagy and senescence) [2].

The aim of this review is to describe how IR may stimulate immunological factors to determine cell fate by multiple pathways by providing an overview of the main key transcription factors that modulate inflammatory gene expression profile after IR exposure. We shall also discuss the cytokine pivotal role in invasiveness and radiation-related fibrosis, and combined radio-immune cancer therapies. Finally, we introduce the future perspectives of a IORT inflammatory response evaluation in understanding inflammatory response induced by a high dose of radiation, in order to identify potential biomarkers that may have a prognostic value for cancer treatment.

### Signalling events in tumour activated by ionizing radiation

In mammalian cells, IR can elicit a multi-layered signalling response by activating many pro-survival pathways that converge to transiently activate key transcription factors (TFs). These include the Nuclear Factor kappa B (NF-kB) and signal transducers and activators of transcription members (STATs) [11,12]. Together, these TFs regulate a wide spectrum of genes involved in inflammation, apoptosis, invasion and angiogenesis processes, contributing to confer tumour cell radioresistance [13,14].

NF-kB is an ubiquitous TF that regulates gene expression profile of more than 200 target genes that have been shown to suppress apoptosis and to induce cellular transformation, proliferation, metastasis, radioresistance and inflammation in a wide variety of tumours [15]. NF-kB has a central role in immune and inflammatory responses because it regulates the expression of pro-inflammatory cytokines and chemokines such as Tumour Necrosis Factor alpha (TNF-α), Interleukine-1 (IL1), Interleukine-2 (IL2), Interleukine-6 (IL6) and Monocyte Chemoattractant Protein-1 for inflammatory cells (MCP-1) [16].

Tumour cells usually express high levels of constitutive NF-kB [17]. IR can activate NF-kB via the Ataxia Telangiectasia mutated protein (ATM) or DNA-PK probably via MEK/ERK/p90 pathway as described by Panta et al. [18]. NF-kB pro-inflammatory responses generally require doses of 7–10 Gray (Gy), but low dose responses have also been observed [19]. The NF-kB activity modulation increases cell growth and survival advantage also through the activation of anti-apoptotic proteins such as BCL-xl, BFL-1/A1, NR13 [20-23]. ATM also phosphorylates p53, which exerts a crucial role following IR-induced DNA damage. As recently described, many IR- induced genes are p53-regulated but there is evidence for a substantial p53- independent IR transcriptional response, where NF-κB plays a contributing role to radioresistance [2]. Like p53, NF-κB activates a variety of genes ranging from cyclins to those involved in lipid signalling and translation [24,25]. Moreover, it was also demonstrated that inhibition of NF-kB activity increased sensitivity of cancer cells to the apoptotic action of different effectors or chemo-radiotherapies [26,27]. Another pathway that plays a key role in regulating the immune response to IR is driven by JAK-STAT signalling. The STAT proteins are considered to be important for cell viability in response to different stimuli such as IR [28,29].

It has been shown that STATs can have a significant role in tumour development and they are included among the potential oncogenes. In normal cells, the activation of STAT proteins is transient (from a few minutes to a few hours). However, constitutive activation of STAT family has been detected in different tumours [30,31]. In particular, STAT1 and STAT3 are very similar proteins (40% identity) that can often be activated by the same extracellular ligand (such as EGF, PDGF or IL-6). They appear to play opposite roles in tumourigenesis: STAT3 is considered an oncogene because it promotes cell survival/proliferation, while STAT1 enhances inflammation and immunity, triggering anti-proliferative and pro-apoptotic responses in tumour cells [31,32].

As recently demonstrated, STAT3 is frequently activated in hematological and epithelial malignancies. This TF induces tumour-promoting inflammation and activates pro-oncogenic pathways (in conjunction”with NF-kB and IL-6) and up-regulates many pro-inflammatory genes such as cyclooxygenase COX-2, IL-1b, IL-6, and IL-8 [33-37]. STAT-3 activation has also been associated with both chemoresistance and radioresistance. STAT-3 mediates these effects through its collaboration with various other transcription factors, including NF-kB, hypoxia-inducible factor-1 (HIF1), and peroxisome proliferator activated receptor-gamma (PPARG). Because of its critical role in tumourigenesis, inhibitors of this factor are being investigated for both cancer prevention and therapy. As described by Aggarwal BB et al. in metastatic breast cancer (BC) cells, chemoresistance is mediated through the up-regulation of anti-apoptotic gene products regulated by STAT-3 [38]. Thus, STAT-3 down modulation can overcome chemoresistance, while its inhibition could promote radiation sensitivity decreasing angiogenesis and cell survival as hypothesized by Kim KW et al. in MDA-MB-231 BC cells [39].

STAT1 plays a dual role in cancer development. Overall, STAT1 induces anti-proliferative and pro-apoptotic genes such as caspases 3, 6, 8, FAS/FASL, p21waf1, c-myc that directly hamper tumour growth. Nevertheless, it has been shown that inappropriate STAT1 activation has also been observed in a variety of neoplastic cells of BC, head and neck squamous carcinoma and others [39,40]. Thus, STAT1 can also favour carcinogenesis and tumour survival. In addition, Khodarev NN et al. showed that ectopically STAT1 increased expression, can induce a radiation resistant phenotype [41]. Furthermore, Hui Z et al. observed that STAT1 down-regulation could significantly increase the radiosensitivity of renal carcinoma cell lines [42]. Thus, its role in acquired radioresistance seems to be based not solely on its transcription activity and needs further investigation.

In conclusion, a number of studies confirm that selective inhibitors of these pro-inflammatory pathways (NF-kB, STAT) could be associated to conventional radiation or chemotherapy [26,30,43] in order to increase their efficiency.

### Cytokine production in response to ionizing radiation

RT has a significant effect on the immune system modulation through the activation of cytokine cascades [44]. The analysis of cancer cytokine signature is therefore a topic of interest in order to understand the roles of cytokines in cancer care [45-47]. Cytokines are produced by tumour cells and tumour-infiltrating lymphocytes (TIL) and can greatly influence cellular radiosensitivity and the onset of tissue complications.

*In vitro* and *in vivo* cells and tissue exposure to IR induces the expression of many cytokines and growth factors such as: TNF-α, IL-1α, IL-1β, IL-6, type I IFN, GM-CSF [44,48-50], IL-4, IL-5, IL-10 [51], IL-12, IL-18 [52], and TGF-β [53].

Cytokine production is *time-dependent*, peaking usually at 4–24 hrs after irradiation with subsequent decrease to baseline levels within 24 hrs to a few days [54]. In all cases, the increase of cytokines and their effects have not yet been investigated at later times such as 72 hr after irradiation.

The balance between pro-inflammatory and anti-inflammatory cytokines is critical in determining a positive or a negative outcome, adverse reaction and resistance to radiation treatment [43]. Many different factors can influence the cytokine profiles produced after radiation exposure. For example, radiation dose, tissue type and the inborn characteristics of tumour cells can influence the local response into a pro- or anti-tumour effect [55,56]. In addition, it is important to realize that *in vivo* and *in vitro* cytokine expression profiles change greatly [57]. Moreover, the pathogenesis of *in vivo* radiation damage has a clear genetic basis, such as polymorphisms in cytokine genes which contribute to the considerable diversity between individuals both in terms of efficacy and adverse reactions [58,59].

Inflammatory reaction induced by RT is mediated by many inflammation-related cytokine genes (e.g., TNF-a, IL-1, IL-6, IL-8, IFN-γ, G-CSF, VEGF, and EGFR), within minutes to hours after an exogenous stress signal [44,50,60]. For example, elevated levels of TNF-α and IL-1 have been found after irradiation of various human or mammalian cells, such as alveolar macrophages or tumour cells [61,62] while an over-production of IL-6 and IL-8 has been described in keratinocytes, fibroblasts and glioma cells after both X-ray or UV exposure [63-65].

Wu CT et al. demonstrated that IL-6 up regulation was positively linked to radiation resistance while its inhibition enhanced the radiation sensitivity in prostate cancer cells [66].

On the other hand, the inflammation response down-regulation is partly due to the short half-life of the pro-inflammatory cytokines and to the production of the anti-inflammatory cytokines, such as IL-4, IL-10, IL-13, and TGF-β [67,68]. These exert an anti-tumour effect, as well as, contributing to tumour immune surveillance escaping.

To date, a few studies have evaluated the cytokine production by cancer cells exposed to high or fractionated dose of IR. It has been suggested that a 20 Gy ablative dose of irradiation produces a more potent immune response than standard fractionation (4 fractions of 5 Gy), promoting the eradication of cancer cells [69].

Recently, Desai S and colleagues have evaluated the cytokines secretion profile of five human tumour cell lines. HT1080 (fibrosarcoma), U373MG (glioblastoma), HT29 (colon carcinoma), A549 (lung adenocarcinoma) and MCF-7 (breast adenocarcinoma), in order to compare their cytokine profiles either before (basal) or after acute (6 Gy) and fractionated doses (3 × 2 Gy) [70]. The authors observed that the secretion of certain cytokines was cell line-specific and that pro-inflammatory cytokines (TNF-α, IL-1β, IL-6), growth factors (PDGF-AA, TGF-α, TGF-β1) and chemokines (fractalkine, IL-8, MCP-1, and IP-10) were highly represented in irradiated conditioned medium (ICM) rather than immuno-modulatory cytokines (IFN-γ IL-2, IL-3, and IL-10). In addition, in all the cell lines studied except for MCF-7 BC, they showed that most of the cytokines increased markedly in a *dose dependent* manner and that the magnitude of such an increase was lower in ICM of tumour cells collected after fractionated IR doses compared to those collected after an acute dose [70].

In a recent study, Belletti B et al. analyzed how normal and mammary carcinoma cell growth and motility are affected by surgical wound fluids (WF) from patients treated with TARGeted Intraoperative radioTherapy (TARGIT). This technique uses a miniature X-ray source that delivers 20 Gy as a single dose of radiation on tumour bed. In this work, using proteomic and phospho-proteomic analysis the authors showed that TARGIT modified significantly the WF protein expression. In particular, after TARGIT treatment, they observed that various proteins including IL-6, MCP-1 and IL-8, and STAT3-drived pathways involved in controlling tumour cell growth and motility, were deregulated [71]. Furthermore, an increase of cytokines produced by Th2 cells (IL-13, IL-4, IL-5) able to induce the differentiation of “tumour-promoting M2 macrophages” expressing anti-inflammatory cytokines, such as TGF-β and IL-10 were described [72,73]. Considering that WF stimulate proliferation, migration, and invasion of BC cell lines [74], this work showed that a high dose of IR delivered by TARGIT could abrogate these processes having an antitumoural effect probably through several growth factors and secreted cytokines.

Cytokines can influence the dose-dependent IR response by their pleiotropic effects, modulating inflammation, invasiveness and fibrosis. For this reason these molecules represent a topic of special radiobiological interest.

### Cytokine-mediated radiation fibrosis

As demonstrated in previous studies, radiation therapy could ultimately culminate in fibrosis [75], characterized by the deposition of collagen and other extracellular matrix components within the stroma and by the presence of atypical fibroblasts.

The IR induced fibrotic tissue remodelling is a multi-cellular process regulated by different cytokines such as TGF-β1, TNF-α, IL-1, IL-4 and IL-13; chemokines such as MCP-1, MIP-1β; angiogenic and growth factors [76-78].

There is substantial evidence that TGF-β1 is primarily involved in normal tissue injury and plays a critical role in the initiation, development, and persistence of radiation induced fibrosis [79]. TGF-β1 belongs to a family of secreted polypeptide growth factors sub-categorized by function, including its three mammalian isoforms (TGF-β1, TGF-β2, and TGF-β3). TGF-β activity is regulated by the latency-associated protein (LAP), and by the latent TGF-β binding protein (LTBP), forming a larger complex called “the large latent complex” (LLC), which can be activated by various physico-chemical treatments or by proteases [80]. In particular, IR increases TGF-ß1 expression and also induces the extracellular activation of the latent complex by proteolytic cleavage in response to the production of reactive oxygen species generated by radiation [81]. TGF-β signals activate the Smad proteins [82], acting as both transduction proteins and transcription factors, able to regulate gene expression of various targets, including procollagen I and III [83].

Moreover, the fibroblasts activation into myofibroblasts is another key step in radiation fibrosis where these cells play an active role in the synthesis and remodelling of extracellular matrix (ECM) components, including collagens. Myofibroblasts are specialized contractile cells that cause aberrant ECM deposition by TGF-β1 activation [84,85]. The resulting increase of matrix proteins, such as collagen and fibronectin, decreases the synthesis of matrix-degrading proteases, and enhances the production of their inhibitors [63,86-88].

In line with these assumptions, Li C et al. demonstrated that BC patients showing high plasma TGF-β1 levels have a major risk of developing post-radiotherapy fibrosis, suggesting its predictive role in IR tumour response [89]. In agreement with these data are those published by Bouquet F and colleagues [90]. These authors demonstrated that TGF-β1 inhibition increases *in vitro* BC radiosensitivity and promotes *in vivo* tumour control by radiation, once again highlighting the relevance of this immune biomarker evaluation during cancer IR treatment. Therefore, TGF-β1 would represent a potential target for molecular therapies designed to prevent or reduce normal tissue injury after IR cancer therapy.

There is some evidence that the IL-4 and IL-13 Th2 cytokines, cooperate with TGF-β to induce fibrosis [91]. IL-4 has long been considered a potent pro-fibrotic mediator nearly twice as effective as TGF-β [92]. This is able to induce the ECM proteins synthesis, collagens and fibronectin. Interestingly, the development of post-irradiation fibrosis is also associated to increased IL-4 production [77,93]. IL-13 shares many properties and functional activities similar to IL-4, as they show common receptor subunits (IL-4Rα), signal transduction pathways and transcription factors (STAT-6) [94]. IL-13 triggers the fibroblasts differentiation into myofibroblasts, induces the production of latent TGF-β1 by macrophages and can also function as its indirect activator by up regulating the expression of LAP cleaved proteins [95,96].

Finally, even the IL-1 and TNF-α pro-inflammatory cytokines have been implicated in fibrosis development. IL-1β is directly up-regulated by radiation and it is known to activate other inflammation-related molecules such as the matrix metalloproteinases (MMPs), a group of zinc-dependent enzymes that regulate or degrade ECM components [97]. Regarding TNF-α, many studies have documented its role in fibrosis development [98,99]. Various strategies involving its inactivation have been designed to protect normal tissue by post-radiation damage. For example, as reported by Przybyszewska M et al. the use of a TNF-α soluble receptor may represent a simple method to partially neutralize TNF-α activity and prevent radiation-induced lung injury [100]. In addition, TNF-α expression leads to the TGF-β1 induction. These two factors, in tandem, regulate IR induced fibrosis acting through multiple mechanisms that need to be largely explored [101].

### Invasiveness, radiation and cytokines

Inflammatory IR response can favour cancer cells invasion, providing a favorable environment for tumour promotion and metastasis [8,10,102-104].

The radiation ability to increase cancer cell invasiveness has been reported for BC, pancreatic, rectal and colon cancer cells [105-107]. IR can alter cell phenotypes which in turn contribute, directly or indirectly, to carcinogenesis and also affects the activity or abundance of proteases, growth factors, cytokines, and adhesion proteins which are involved in tissue remodelling [108].

Both IL-8 and IL-6 are involved in IR inflammatory response, enhancing cancer cell invasiveness [109].

IL-8 is a member of the CXC chemokines superfamily and has a wide range of pro-inflammatory effects. It was initially described as a neutrophil and lymphocyte chemo-attractant [110] but has subsequently been identified as a pro-angiogenic agent in a wide range of human malignancies [111,112]. For example, as reported by De Larco JE et al. in BC cells the metastatic phenotype is strongly correlated with IL-8 expression, suggesting it as a prognostic metastatic biomarker [113]. This chemokine is probably up-regulated in a *dose dependent*-manner, as described by Singh RK et al. in human melanoma cells and by Meeren AV et al. in endothelial cell lines, in tandem with IL-6 production [114,115].

IL-6 is one of the most important pro-inflammatory cytokine but it has also been considered to have an anti-inflammatory role for its ability to induce IL-1 and TNF-α antagonists [68]. IL-6 has been reported to be increased in a variety of tumours, contributing to aggressive tumour growth and resistance to treatment [116-119]. Circulating IL-6 levels are positively associated to a clinical tumour stage, lymph node infiltration, and the number of distant metastases in BC patients [120,121]. In turn, in BC cells it has been described that the IL-6 JAK/STAT3 pathway could promote BC progression, metastasis, resistance to treatment [122] and, at the same time, IL-6 induced through STAT3 can then activate IL-6/STAT3 signalling in neighbouring cells.

In addition, the IR induced IL-1β expression can also favour cancer cell invasion. For example, in BC patients, elevated IL-1β plasma levels have been shown to persist for a few weeks after radiotherapy [123]. IL-1β is involved in the enhancement of BC cell invasion induced by radiation. As described by Paquette B et al., this cytokine can also enhance cancer cell invasion acting as a chemo-attractant agent for MDA MB-231 BC cells [124]. The authors suggested that the effect of IL1β on these BC cell invasiveness involves the elevation of MMP-9 production, the induction of COX-2 expression and the prostaglandin E2 (PGE2) biosynthesis.

Moreover, some preclinical models suggest that radiation activated TGF-β could contribute to metastasis inducing the appearance of mesenchymal characteristics [125-127]. Several lines of evidence have led researchers to link this morphological shift during carcinogenesis to the process of epithelial to mesenchymal transition (EMT), which is an important step in cancer invasion and metastasis [128-130]. In particular Zhou YC et al. suggested that TGF-β mediated EMT plays a critical role in enhancing the migratory and the invasive capabilities of the IR induced cancer cells [131]. The complexity of the radiation effects mediated by TGF-β will require further study to determine whether it plays a proximal role in promoting radiogenic carcinogenesis.

These data advocate the need of further clarification on the ability of radiation to increase the invasiveness of cancer cells, probably mediated by immunological factors.

### Radiation and inflammation combined cancer therapies

Nowadays, combinatorial anticancer therapy is an established clinical practice. This is based on the principle that stand-alone, chemo or radio-therapeutic regimens are generally unable to control neoplastic lesions, whereas combining therapeutic agents with dissimilar action mechanisms potentially results in synergistic anti-neoplastic effects [132-135].

IR leads to the activation of several immunological proteins and TFs modulating the expression of numerous immune mediators that may promote cancer development. Thus, targeting the IR induced inflammatory signalling pathways offers the opportunity to improve the radiation therapy clinical outcomes by enhancing radiosensitivity [103,136].

For example, as described in literature, the disruption of NF-kB signalling could be associated to conventional cancer therapies in order to increase their efficiency [137], specifically to improve treatment programmes for chemo-resistant and or radio-resistant cancers. Different approaches to inhibit NF-kB activity are proposed in various models [138-140]. It has been observed that the IKB over-expression sensitizes human glioblastoma, fibroblast and intestinal epithelial cells to radiations [141]. In recent years the NF-κB inhibition by synthetic compounds as well as nutraceuticals factors has been approved for tumour radio-sensitization [142]. In particular the use of the herbal medicine curcumin has become a useful approach due to the anti-inflammatory properties in conjunction with low toxicity risk. Furthermore, curcumin has been shown to down-regulate the NF-kB expression and STAT-3 phosphorylation [143]. Another relevant NF-kB mediated-response to IR DNA damage is induced by the activation of the autocrine TNFα-TNFR1 signalling able to cause IkB proteasomal degradation and the final NF-kB activation. TNF-α is both an inductor and a gene target gene of NF-kB [29,30]. Its induction could create a loop which amplifies the effects of radiation. Thus, the NF-kB inhibition results in a complex network, either an increased apoptosis of irradiated cells, or a lower TNF-α production decreasing the therapeutic effects of radiations [137,144,145].

Radiation is also known to induce inflammation through COX-2. COX is the key enzyme required for the conversion of arachidonic acid to prostaglandins [146,147]. It is a central enzyme in the inflammatory response, its activity in cancer cells can be directly stimulated by NF-kB after radiation exposure or indirectly through some cytokines activity, such as IL-1β [124,148]. The COX-2 over-expression has been shown in patients with various types of cancers. In particular COX-2 up regulation is associated to higher tumour grade and distant metastases in BC [148,149]. This protein has assumed an important role as therapeutic target for anticancer and anti-inflammatory therapies because its inhibition by drugs such as ascoxibs, celecoxib, and SC-236, represents a radio-sensitization strategy [150,151].

In mammalian cells, IR can elicit the activation of multiple targets and key TFs, including the above mentioned STAT3, representing a promising therapeutic target for preventing inflammation-mediated cancers [39]. In the past few years several new molecules inhibiting STAT3 (such as small interfering RNAs, oligonucleotides, small molecules) were tested both *in vitro* and *in vivo* approaches [152-155]. Stable transfection with shRNA against STAT3 results in enhanced radiosensitivity of human squamous carcinoma (A431) cells [156]. Targeting of STAT1 might be a potential strategy to sensitize cancer cells. Zhan JF and colleagues, provided the first evidence that STAT1 signalling contributes to radioresistance in BC initiating cells, revealing STAT1 as a promising target to reduce radioresistance [157]. For instance, Hui Z et al. observed that the STAT1 down-regulation sensitizes renal cell carcinoma (RCC) to chemotherapy and radiotherapy [42].

In addition, the previously mentioned cytokines IL-1β, TNF-α, IL-8, IL-6 or TGF-β can influence the response to IR, inducing inflammation, cancer cells invasiveness and fibrosis in irradiated tissues, encouraging the hypothesis of the use of specific inhibitors or drugs able to manipulate cytokine pathways, in order to improve radiation research and therapy. For example, Salem K and colleagues have recently demonstrated that the combined treatment with the two chemotherapeutic drugs dexamethasone (Dex) and bortezomib (BTZ) attenuates paracrine IL-6 secretion from irradiated stromal cells, contributing to myeloma cell death and the inhibition of therapy resistance [42]. The authors suggested that the Dex and BTZ combined treatment may effectively eradicate myeloma cells in their native bone marrow microenvironment in patients undergoing oxidative stress-induced therapy, since the Dex treatment effectively reduces IL-6 secretion from irradiated stroma and BTZ has been shown to be effective in blunting IL-6-mediated survival signalling in myeloma cells [42]. It will be of particular interest to analyze the results of multiple clinical trials that are currently evaluating the safety and the anti-neoplastic profile of radio-immunotherapeutic based regimens in cancer patients. Hence, the use of specific inhibitors and the manipulation of cytokine pathways, involved in cancer cell proliferation and metastasis, are very important to improve radiation therapy [158,159]. Pre-clinical studies have demonstrated that combining radiotherapy with immune stimulation can induce an anti-tumour immunity, enhancing cell death [132-135]. In order to optimize and personalize radiation-therapy treatment for each patient, a multi-parametric approach would be useful to identify several potential targets that may affect radio-response.

### Future perspectives of a IORT inflammatory response evaluation

Intraoperative radiation therapy (IORT) is particularly appealing to patients and physicians, because the procedure is fast, convenient, spares normal tissue to a considerable extent and is able to solve some clinical problems, like the integration with chemotherapy with respect to conventional RT [160]. IORT differs from conventional RT since a large dose in a single fraction during surgery is delivered. The BC IORT treatment, according to specific eligibility criteria, may be performed using two different protocols: *exclusive* with the provision of a single radiation dose of 21–23 Gy, corresponding to the administration of the entire sequence of a conventional adjuvant RT or as an anticipated *boost* of 9–12 Gy, followed by conventional external RT treatment [161,162]. This potentially eliminates repopulation of residual tumour cells that may occur during wound healing before post-operative radiotherapy can begin. IORT with electrons was associated with about the same number of distant metastases and deaths as external RT, showing that distant disease control and overall survival are much the same in two treatment groups, at least in the short term. The continued active follow-up of patients in medical trial will allow to reassess the safety of IORT with electrons on the development of distant metastases and death in the long term [163,164].

Most radiobiological studies on cell lines have been performed in the dose range 1–8 Gy where cellular radiation effects, including clonogenic inactivation and survival can be studied properly. However, some evidence suggests that high single doses used in IORT may produce different effects from those seen after conventional fraction sizes [164,165].

Few papers describe the role of inflammatory response to high doses of IR, such as those used in IORT treatments, rendering in this field the necessity of being explored and clarified. Some evidence suggests that high single-dose regimens are more efficient than low-dose regimens to trigger both innate and adaptive anti tumour immunity [71]. For example, in the above mentioned study by Belletti *et al*. was demonstrated that TARGIT treatment modified significantly the protein expression of the WF [71]. In addition, the WF from TARGIT-treated patients showed a modified expression of certain cytokines, and loss of the ability to induce the activation of some intracellular signal transduction pathways. As above described, WF stimulate proliferation, migration, and invasion of BC cell lines, showing that a high dose of IR delivered by TARGIT could abrogate these processes producing an antitumoural effect probably through several growth factors and secreted cytokines [74]. It opens a novel avenue for identifying new molecular targets and testing novel therapeutic agents depending also to high dose delivered that need to be further explored considering limited but encouraging available data on this topic [166,167].

In order to highlight the molecular mechanisms involved in the response/resistance to IORT treatment, our research group is performing the study of IR effects on BC cells subjected to high dose treatment modalities and their correlation with genetic background and molecular gene expression. Regarding the inflammatory response, in particular, we are focusing our attention on immunogenic factors induced by a high dose of radiation, in order to identify potential biomarkers that could influence radio-resistance or down regulate BC cells invasiveness, as well as markers that may have a prognostic value for cancer treatment.

## Conclusion

**Figure 1:**
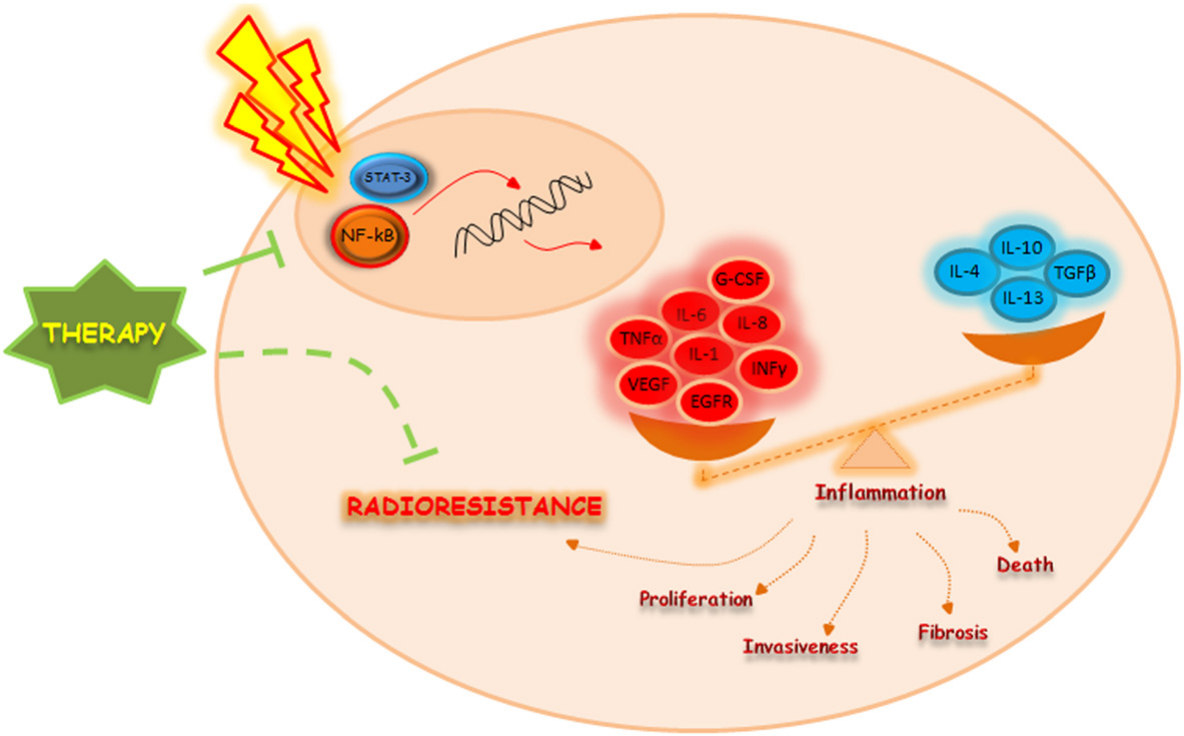
**Immunological response to IR.** The figure displays how IR could stimulate key transcription factors modulating inflammatory gene expression profile and cytokines involved in invasiveness and radiation related fibrosis. Targeting Nf-kB and STAT-3 IR activated, could offer the opportunity to improve radiation therapy by enhancing radiosensitivity.

RT has extensively been employed as a curative or palliative intervention against cancer throughout the last century, with a varying degree of success. IR activates complex cross-linked intracellular pathways able to define cell fate, determining the outcome between survival and death regulating several factors involved in inflammation, DNA repair, cell survival or death (summarized in Figure 1) [2]. The immune system plays a pivotal role controlling tumour development, suppression or tumour progression [8]. This review represents an overview, of the most recently available data regarding the main networks activated after IR exposure, cytokines pivotal roles in invasiveness and fibrosis radiation-related and radio-immune combined cancer therapies updates.
